# Effects of dance-based interventions on body image and self-worth in overweight and obese individuals: a systematic review

**DOI:** 10.3389/fpsyg.2026.1836311

**Published:** 2026-07-29

**Authors:** Luisa Heyn, Tina Schwender, Helena Rudi, Mara Köhntopp

**Affiliations:** 1 Department of Sport Pedagogy and Didactics, Institute of Sport Science, Johannes Gutenberg-University, Mainz, Germany; 2 Department Health and Sport Sciences, TUM School of Medicine and Health, Technical University of Munich, Munich, Germany

**Keywords:** interviews, mixed methods, psychosocial health, qualitative methods, quantitative methods, questionnaires

## Abstract

**Background:**

Emerging theoretical frameworks and empirical findings indicate that dance interventions may positively influence body image and self-esteem in individuals with obesity. This systematic review aims to identify and synthesize studies examining the effects of dance on body image components (e.g., body perception) and facets of self-esteem (e.g., self-acceptance) in this population. Specifically, the review addresses: (a) the current state of evidence on the impact of dance interventions on body image and self-esteem in individuals with obesity, and (b) the intervention characteristics that contribute most effectively to improvements in these psychosocial outcomes.

**Methods:**

A systematic literature search was conducted across multiple online databases, including peer-reviewed journals in English and German. Studies were eligible if they met the following criteria: (1) intervention, clinical, observational, longitudinal or case study designs; (2) assessment of body image and/or self-esteem constructs or components; (3) inclusion of participants with obesity; and (4) dance as a central intervention element. Three independent reviewers evaluated study eligibility following PRISMA guidelines and assessed methodological quality.

**Results:**

The ten included studies represent diverse populations and intervention formats, spanning children to adults and encompassing a variety of dance-focused approaches. Due to substantial heterogeneity in study design, duration, and methodology, findings were analyzed using the *Wheel of Dance* framework. Interventions targeted the following components: group cohesion, culture, and history (1 study); aesthetic fitness and physical health (2 studies); connection and connectedness (1 study); flow and mindfulness (5 studies); and aesthetic emotions and imagination (1 study). Overall, dance interventions were associated with positive effects on body image, self-esteem, and psychosocial well-being, independent of weight change. Creative dance formats particularly fostered emotional expression, social connectedness, and improved body awareness. Considerable variation existed across studies in terms of intervention design, duration, methodological rigor and quality.

**Conclusion:**

The findings of this review suggest that dance may represent a potentially promising complementary intervention for addressing psychosocial health in individuals with obesity. However, given the limited evidence base and the heterogeneity of study designs, these findings should be interpreted with caution. Further methodologically rigorous studies are required, particularly those with a stronger focus on psychosocial constructs.

**Systematic review registration:**

https://www.crd.york.ac.uk/PROSPERO/view/CRD42024604272, identifier CRD42024604272.

## 1 Introduction

The engagement with one’s own body is historically, culturally, and socially embedded (Cash, 2004; Schilder, 2013). In contemporary society, however, the relationship with the body is ambivalent—a dynamic further amplified by the use of digital media, particularly social networks. These platforms facilitate both self-representation and social comparison (Fardouly et al., 2015; Krayer et al., 2007; Peter et al., 2012; Tiggemann and McGill, 2004; Vogel et al., 2014). On one hand, movements such as body neutrality promote a partially appreciative or at least non-judgmental stance toward one’s own body (Aubrey et al., 2024; Merino et al., 2024; Seekis and Lawrence, 2023); on the other hand, they exist in tension with established societal and media-driven beauty and performance norms (Höger, 2024; Jensen et al., 2022; Pürgstaller, 2023; Rudi and Theis, 2024; Ruin, 2023; Ruin and Giese, 2018; Schemer, 2007). This dichotomy of conflicting body discourses places substantial demands on individual body perception. These challenges are particularly pronounced in individuals with obesity, whose bodily experience is often shaped by stigmatization, shame, and psychosocial burdens (Legault and Sago, 2022; Seekis and Lawrence, 2023).

### 1.1 Body image as a multidimensional construct

Body image, understood as the subjectively perceived representation of one’s own body size, shape, and form (Pollatou et al., 2010; Slade, 1994), occupies a central role in this context. It emerges from visual, aesthetic-sensory, and cognitive engagements with both the body and the environment (Kappert, 1990). Unlike objectively measurable parameters such as body weight or height, body image is closely linked to emotional evaluations, individual interpretations, and socially constructed meanings (Pollatou et al., 2010; Schemer, 2007). Accordingly, body image is described as a multidimensional construct. Vocks et al. (2018) differentiate between a perceptual component (body-related perception), a cognitive-affective component (individual evaluation of the body), and a behavioral component (body-related behavior). In addition, Cash and Pruzinsky (2002), identify an evaluative component, reflecting the discrepancy between real and idealized self-perception, as well as a dysfunctional investment component, which captures the importance of appearance for an individual’s thoughts and behaviors.

Thus, body image cannot be considered solely within the framework of individual developmental processes (Fardouly et al., 2015; Krayer et al., 2007; Peter et al., 2012; Tiggemann and McGill, 2004; Vogel et al., 2014). A positive body image is regarded as a protective factor, as it is associated with higher quality of life, more stable self-esteem, and better mental health outcomes (Annesi, 2017). As a subjective perception, evaluation, and set of behaviors (Denninger, 2018), body image can exert both supportive and detrimental effects on self-esteem (Ouyang et al., 2020). Consistently, studies demonstrate a positive correlation between body satisfaction and global self-esteem (Tewatia, 2017), whereas negative body perception is frequently associated with lower self-esteem and increased depressive symptoms (Nazia et al., 2022; Satwik et al., 2024).

### 1.2 Dimensions of self-esteem

**FIGURE 1:**
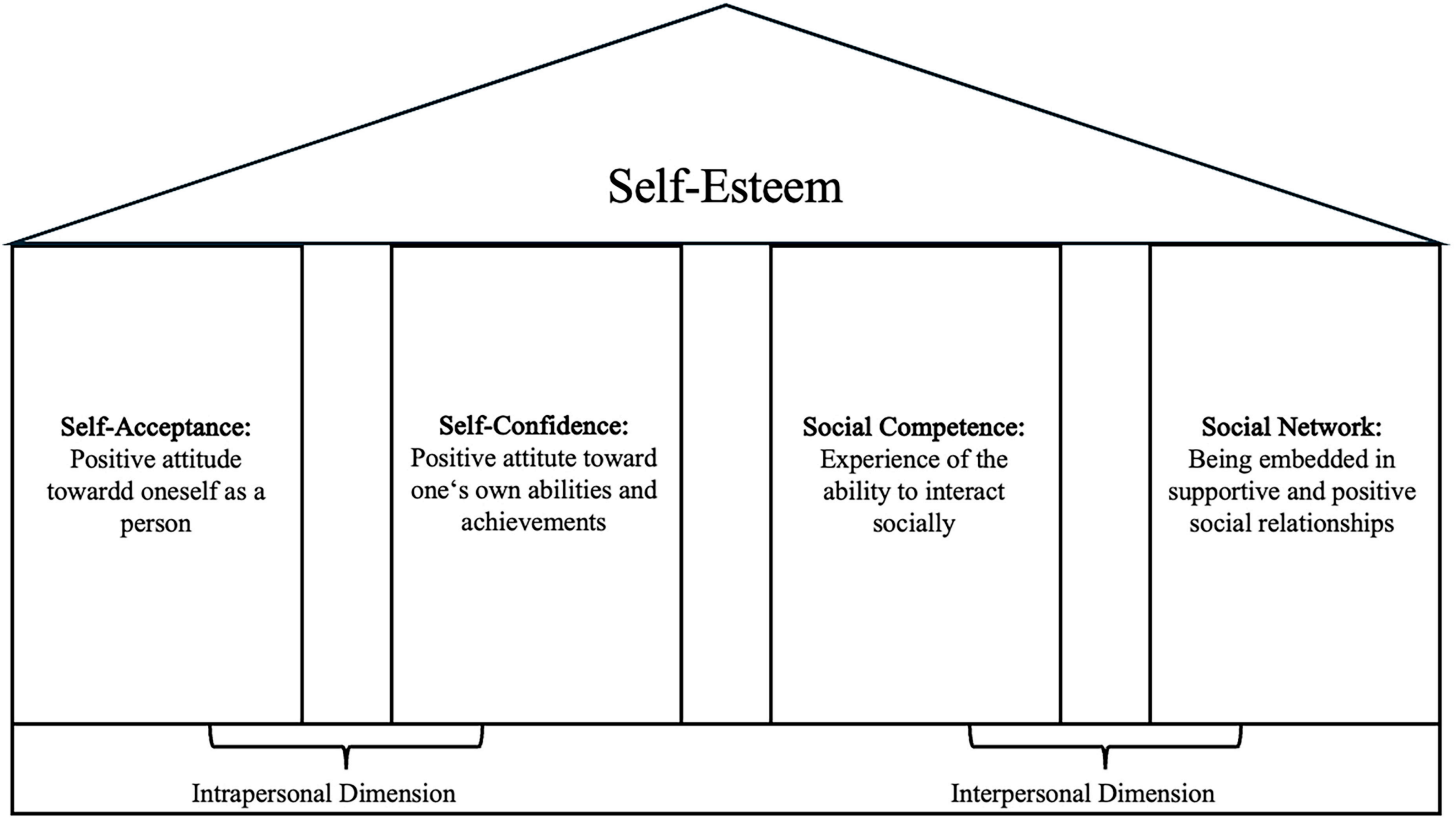
Four pillars of self-esteem according to Potreck-Rose and Jacob (2012) (simplified illustration by the authors).

Self-esteem is a multifaceted psychological construct closely linked to emotional well-being, motivation, and social interaction, and describes the subjective evaluation of one’s own worth (Dorsch, 2021; Schwender et al., 2018). A differentiated understanding is provided by the “four pillars of the self-esteem system” model by Potreck-Rose and Jacob (2012, 2021) (see Figure 1), which distinguishes between self-acceptance and self-confidence. Social competence and social networks are classified within the interpersonal dimension. While self-acceptance refers to a fundamental attitude toward oneself, self-confidence relates to the evaluation of one’s own abilities and performance. Social competence encompasses the ability to shape social relationships, whereas the social network refers to integration into supportive social relationships. Self-esteem is a dynamic construct influenced by affective states (Baumann, 2020; Potreck-Rose and Jacob, 2012).

Self-esteem derives from self-concept-related knowledge and the evaluation of one’s own person (James, 2018; Rosenberg, 1979; Rudi, 2021; Schwender et al., 2018) and influences cognition, motivation, and behavior. Social feedback plays a central role, as the self develops through interaction with the social environment (Cooley, 1983). High self-esteem is associated with greater well-being and more stable social relationships, whereas low self-esteem may be linked to insecurity and psychosocial burden (Orth et al., 2012; Rudi, 2017). Self-evaluative experiences may manifest as both relatively stable trait-like characteristics and situation-dependent state fluctuations (Heatherton and Polivy, 1991; Marsh and Craven, 2006; Segal, 1988).

In addition to the distinction between intra- and interpersonal dimensions, a differentiation is made between global self-esteem (overall evaluation of the self) and domain-specific expressions. Interpersonal dimensions relate to specific areas of the self-concept, such as academic, social, or physical domains (Shavelson et al., 1976), as well as domain-specific constructs such as the dance-related self-concept (Rudi, 2021).

While the terms self-worth and self-esteem are frequently used synonymously in the international literature, a conceptual distinction is made in the present work. Self-worth refers to a fundamental, largely performance-independent experience of one’s own value and is therefore conceptually close to self-acceptance. In contrast, self-esteem refers more to the evaluative assessment of one’s abilities, competencies, and performance, and is thus more closely related to the concept of self-confidence. This distinction is particularly relevant in the context of obesity, as evaluation of the body or performance may influence self-esteem without necessarily affecting an individual’s fundamental sense of self-worth. Due to this conceptual alignment, particularly in sensitive contexts such as self-worth in individuals with obesity, the term self-worth is used throughout the present review (Ackerman and Lucas, 2025).

### 1.3 Psychosocial significance of body image and self-worth in individuals with obesity

In individuals with obesity, a condition characterized by elevated body fat mass (Ipsen et al., 2016; Vekic et al., 2019), body image represents a particular challenge (Kinzl, 2016; McIntosh et al., 2016). Affected individuals often perceive their bodies as unattractive, which can negatively impact self-worth (Friedman et al., 2002; Schwartz and Brownell, 2004). Additionally, distorted perceptions of one’s body size can influence behavior and mental health (Gesell et al., 2010). This discrepancy between the idealized and actual body shape leads to dissatisfaction and diminished self-worth (Neumark-Sztainer et al., 2006; Sarwer et al., 2005; Stice, 2001, 2002). A negative body image is also considered a central risk factor for eating disorders (Striegel-Moore and Franko, 2002), whereas improvements in body image can positively influence eating behaviors (Carraça et al., 2012).

Body image concerns therefore affect psychosocial health, quality of life, and social participation (Hebebrand and Herpertz-Dahlmann, 2009; Incledon et al., 2011; Neumark-Sztainer et al., 2006; Wardle and Cooke, 2005; Zametkin et al., 2004). Individuals with obesity frequently experience stigmatization, social withdrawal, interpersonal stress, and barriers to participating in physical activity programs (Chu et al., 2019; Cooper et al., 2008; Dunker and Claudino, 2018; Matz et al., 2002; Schwimmer et al., 2003; Steptoe and Butler, 1996).

These psychosocial burdens can particularly act as barriers in the context of weight reduction (Cooper et al., 2003). Although fitness centers are commonly recommended as a means to reduce body weight, these environments often promote a specific body ideal, leading many individuals with obesity to feel uncomfortable and avoid such social settings (Jiménez-Loaisa et al., 2020; Thedinga et al., 2021). At the same time, studies indicate that physical activity in a group setting can reduce depressive symptoms and improve both body image and self-worth (Heath et al., 2022; Milne-Ives et al., 2025). This highlights the importance of socially supportive, inclusive, and appreciative physical activity opportunities for individuals with obesity.

### 1.4 Dance as an embodied and aesthetic form of expression

Dance represents a unique form of physical activity that, through conscious awareness of individual bodily limits, fosters the reciprocal relationship between movement and body consciousness (Deasy, 2002; Deasy et al., 2002; Kirsch, 2005; Klinge, 2017; Rudi, 2021). The integration of cognitive, emotional, and bodily processes allows for diverse forms of expression and has positive effects on body image, self-worth, and subjective bodily experience (Bachmann, 2017; Lowinski, 2007; Schwender et al., 2018; Trautmann-Voigt, 2003; Tschacher et al., 2014). In dance, the body is not merely an object but an active, sensorially experiencing subject, enabling the engagement of deeper affective-cognitive structures (Fong et al., 2024). This quality is grounded in the aesthetic-cultural, creative, and non-performance-oriented structure of dance, which provides the body with diverse expressive possibilities (Steinberg and Rudi, 2024).

Central to dance is the embodied experience as a dynamic interaction between thoughts, bodily experience, and the environment (Schwender et al., 2025). As humans experience the world with a body-anchored mind and through their senses, bodily experience assumes a pivotal role (Kosmas and Zaphiris, 2018). Learning thus always occurs through and with the body (Singh and Narayanan, 2021). This perspective is also reflected in aesthetic education, which extends beyond mere reception of information to include processes of touching, moving, and engaging (Brandstätter, 2018). Aesthetic experiences therefore possess transformative and pedagogical potential (Zirfas and Klepacki, 2013).

The uniqueness of dance lies in its diversity and openness: it integrates music, movement, and self-expression, thereby engaging multiple senses (Kirsch, 2005). The goal is not technical perfection but the impact of creative and exploratory action (Laban et al., 2021). Dance-specific movements also create spaces for processing emotional experiences beyond verbal communication (Huschka, 2002). Through creative forms of expression, a deeper connection to the sensing body is facilitated, allowing bodily sensations, emotions, and implicit knowledge to be perceived more consciously (Zitt, 2008). Moments of disruption play a central role: they dissolve conventional patterns of perception and expression, enabling the emergence of new experiential spaces (Obermaier, 2024).

### 1.5 Dance as a physically-cognitive and psychosocial medium

Dance is not a homogeneous concept but encompasses diverse forms with specific effects, objectives, and physical-cognitive demands (Christensen et al., 2021). They emphasize that the health-related effects of dance appear heterogeneous precisely because no unified definition exists. Different dance styles vary substantially in physical intensity, social interaction opportunities, and cognitive requirements. To systematically classify these variations, Christensen et al. (2021) propose six cross-style components, which are used here as an analytical framework:

(1) Music and rhythm: Dance integrates movement with music and emotion. While music is not strictly necessary, recreational dance usually occurs with or to music or rhythms (Jola et al., 2011; Stevens et al., 2009). The close coupling of auditory and motor systems explains why music can structure and synchronize motor processes (Kornysheva et al., 2010; Thaut et al., 1999; Zatorre et al., 2007). People also move intuitively to music (Bernardi et al., 2018), which can positively affect stress reduction, immune function, emotion regulation, and social bonding (Fancourt et al., 2014; Quiroga Murcia et al., 2009; Sachs et al., 2015; Trehub et al., 2015; Welch et al., 2014).

(2) Group cohesion and culture: Dance has strong social bonding potential, fostering shared movement experiences, social interactions, and emotional resonance. Emotional expression can be more pronounced in dance than in other sports, supporting interpersonal connectedness (Freedberg and Gallese, 2007; Shafir et al., 2016). According to the Polyvagal Theory (Porges, 1991), social interactions such as affective resonance and touch can positively influence mental and physical well-being. Dance also strengthens group-dynamic competencies such as impulse control, respect, and cooperation (Lobo and Winsler, 2006; Staiano et al., 2018) and provides marginalized or excluded individuals with opportunities for social participation (Christensen et al., 2021). Furthermore, dance creates spaces for trust, self-expression, and self-perception (Heinrichs et al., 2003; Keysers et al., 2004; McGlone et al., 2014).

(3) Aesthetics, fitness and technique: Dance integrates cognitive, coordinative, and physical demands. Complex movement sequences, spatial orientation, and multisensory integration engage both motor and executive functions, particularly in technically oriented dance forms (Christensen et al., 2017; Orgs et al., 2013). Empirical evidence indicates positive effects on blood pressure, sleep, physical performance, quality of life, body awareness, and self-image, even among individuals with obesity (Allet et al., 2017; Conceição et al., 2016; Mangeri et al., 2014; Muller-Pinget et al., 2012; Serrano-Guzmán et al., 2016).

(4) Connection and connectedness: A central feature of dance is the experience of connection, with oneself, music, dance partners, or the group (Bernardi et al., 2018; Christensen et al., 2017; Rudi et al., 2022). This subjective sense of connectedness correlates with positive evaluations of the dance experience (Bernardi et al., 2018; Duberg et al., 2016) and contributes to enhanced physical, psychological, and social functioning (Eyigor et al., 2009; Mavrovouniotis et al., 2010; Zhang et al., 2013).

(5) Flow and mindfulness: Dance promotes flow experiences, which are associated with increased well-being (Gore, 1997; Jeong, 2012). Experiencing “groove” intensifies immersion in the activity (Bernardi et al., 2018), and the targeted use of imagery can further enhance this experience (Jeong, 2012). Due to its physical-cognitive demands, dance also parallels mindfulness practices, promoting body awareness, present-moment focus, and self-regulation (Marich and Howell, 2015), which positively impact emotion regulation, empathy, and life satisfaction (Beddoe and Murphy, 2004; Shapiro et al., 1998; Vago and Silbersweig, 2012).

(6) Emotions and Fantasy: As an embodied art form, dance integrates imagination, metaphor, and sensory imagery (Goldschmidt, 2002), encompassing visual, kinesthetic, musical, metaphorical, and emotional dimensions (Coker et al., 2015; Golomer et al., 2008; Jakubowski, 2020; Shafir et al., 2016; Tsachor and Shafir, 2017). These processes activate complex motor and emotional circuits (Carlson, 2013) and support self-expression and emotion regulation (Koch et al., 2014). A key mechanism is interoception (Christensen et al., 2016; Schirmer-Mokwa et al., 2015), the conscious perception of internal bodily states, which is linked to self-awareness, resilience, and agency (Tsakiris, 2017; Werner et al., 2013).

Dance offers particular potential for addressing body image, as body image encompasses cognitive, affective, and often implicit self-attributions. Dance can act as a sensorial-reflective practice, initiating processes of change (Vocks et al., 2006, 2018). For individuals with obesity, whose body image is often shaped by societal stigma, shame, and reduced self-efficacy (Legault and Sago, 2022; Seekis and Lawrence, 2023), dance provides a safe experiential space beyond normative performance expectations.

This systematic review provides an overview of studies examining the effects of dance-based movement programs on body image and self-worth among individuals with obesity. Qualitative, quantitative, and mixed-method studies are included to encompass diverse forms of evidence (Dixon-Woods et al., 2005). The review addresses the following research questions: (a) What is the current state of knowledge regarding the effects of dance interventions on body image and self-worth in individuals with obesity? (b) Which specific intervention characteristics demonstrably contribute to improvements in body image and self-worth in individuals with obesity?

## 2 Methods

This systematic review was conducted following the PRISMA guidelines (Moher et al., 2015; Page et al., 2021), which provide structured checklists and recommendations to ensure transparent reporting, methodological rigor, and reproducibility.

### 2.1 Search strategy

The systematic literature search was conducted by the first author (LH) in the following electronic databases: EBSCOhost, PubMed, OLC Database, ProQuest, Educational Research, SURF, PubPsych, Web of Science, BASE, SpoNet, BEME, Cochrane Library, ERIC via IES, and Livivo. The selection of databases was guided by the aim of capturing an interdisciplinary spectrum spanning medicine, psychology, education, and sport science. No restrictions were applied regarding publication year; both English- and German-language studies were included.

The search strategy was based on three core concepts: (1) self-worth and/or body image, (2) dance-related interventions, and (3) target population with overweight or obesity. These concepts were combined Boolean operators (AND/OR) and expanded trough synonyms and truncations to ensure a comprehensive identification of relevant literature.

The search terms were developed collaboratively by the authors (LH, TS, HR) and were as follows: (Fettleibig* OR Adipos* OR Adipös* OR Obesit* OR Übergewicht* OR overweight OR obese) AND (Körperbild* OR “Body image*” OR Selbstwert* OR self-esteem*) AND (Sport* OR Bewegung* OR Training OR Physisch* OR Übung* OR Aktivit* OR Movement* OR Motion* OR Physical* OR Tanz* OR Tänz* OR Tanztherapie* OR Danc* OR “Dance Movement Therap*”). The database search commenced in October 2024, with the final update conducted on February 28, 2025. The review protocol was preregistered on PROSPERO^1^ under the registration number CRD42024604272.

### 2.2 Inclusion and exclusion

The defined inclusion and exclusion criteria are summarized in Table 1.

**TABLE 1:** Inclusion and exclusion criteria.

Inclusion criteria	Exclusion criteria
Intervention studies, clinical trials, observational studies, longitudinal studies, case studies	Combined programs
Assessment of aspects of body image and/or self-worth	
Individuals with obesity or overweight	
Dance as the intervention content, including dance and movement therapies as well as fitness dance such as Zumba, aerobic dance, etc.	
Language: English and German	
Published as peer-reviewed journal article	

Only original research articles published in peer-reviewed journals were included to ensure a high scientific standard regarding content, structure, language, and methodological rigor. Multimodal physical activity programs in which dance was merely one of several equivalent or optional activities, such as walking, cycling, yoga, or strength training, were excluded. This exclusion was applied for methodological reasons, to allow effects to be clearly attributed and the specific impact of dance on body image and self-worth to be validly assessed. In programs without a clear emphasis on the individual components, this attribution is not possible (Moher et al., 2015; Page et al., 2021). Studies were included if dance was combined with lifestyle interventions, such as nutritional counseling or psychological support, if dance was explicitly defined as the central movement-based intervention.

### 2.3 Study selection procedure and risk of bias assessment

After completing the database search, all identified references were screened in a multi-stage process. Two reviewers (LH, MK) independently examined the titles of all extracted studies. Potentially eligible articles were included in the abstract screening phase. In cases of uncertainty, studies were initially included to allow a more informed decision at the abstract level.

The abstracts were also independently assessed. If the information provided was insufficient to determine eligibility with confidence, the full text was advanced to the next review stage.

The full texts of potentially relevant studies were then evaluated in detail against the predefined inclusion and exclusion criteria. Reasons for exclusion at the full-text stage were systematically documented (LH, TS). In addition, the reference lists of the included studies (LH) were checked to identify further relevant publications.

Discrepancies at any stage of the review were resolved through discussion among the authors (LH, TS, MK) to reach consensus. In cases of persistent disagreement, a fourth reviewer (HR) was consulted.

To ensure transparency and reproducibility, the assessment of study eligibility was conducted independently by two reviewers (LH, TS). Ratings were based on the predefined criteria using the categories “include,” “exclude,” and “uncertain.” A total of 26 full texts were independently assessed. In 21 cases (80.8%), there was agreement between the reviewers. The remaining five cases were resolved through discussion and consensus with the involvement of a third reviewer (HR). This procedure aligns with PRISMA guidelines and serves to enhance transparency, reproducibility, and methodological rigor (Moher et al., 2015; Page et al., 2021).

### 2.4 Data exclusion

For this review, a structured data extraction protocol was developed. Based on this protocol, the following information was systematically extracted from the included full texts: (1) General study information: authors, year of publication, country, study design, and data collection period; (2) Participant characteristics: age, sex, BMI, sample size, and specific characteristics (e.g., physical or cognitive comorbidities); (3) Intervention characteristics: content of the dance intervention, duration, frequency, delivery modalities, presence of a control group, and details of the control condition; (4) Assessment methods; and (5) Results and key findings. Data extraction was conducted by the first author (LH).

### 2.5 Data analysis and synthesis

Due to the conceptual and methodological heterogeneity of the included studies, as well as the limited comparability of outcome measures, a statistical meta-analysis was not conducted. Instead, a theory-driven narrative synthesis was performed. Study results were systematically analyzed and presented according to the components of the *Wheel of Dance* defined by Christensen et al. (2021). This framework served as an analytical structure for categorizing intervention focuses and potential mechanisms of action.

For clarity, two summary tables were created: Table 2 summarizes general study characteristics according to the *Wheel of Dance* components, while Table 3 provides detailed information on the specific intervention content.

**TABLE 2:** Framework conditions of qualitative, quantitative, and mixed-method studies.

Author, year, sample size	Participant type, BMI, country	Intervention		Study design	Assessment methods for body image and self-worth	Outcomes on body image and self-worth
		Intervention content, control group	Duration, frequency			
Group cohesion and culture						
Azevedo et al. (2013); *N* = 43 (40 families)	Girls aged 8–11; BMI not reported; California	Mexican folklore dance; no control group	2-year after-school dance program, five times per week; 1.5–3 h/session	Part of RCT ECHALE/ethnographic study	Interviews (parents and girls)	Parents reported increased self-worth and self-confidence, better attitudes, less shyness, improved social bonds
Aesthetic fitness and technique						
Akyilmaz et al. (2023); *N* = 150	White Caucasian women; 30.29 ± 5.2 years; BMI > 30 kg/m^2^; Helsinki	Zumba; 2 control groups (Walking and general control)	8 weeks; five times per week; session duration 30 min at start, 60 min at end	Randomized controlled experimental study; pre-post test design	Body Image Scale (BIS) (Hovardaoǧlu, 1992); three-factor eating questionnaire—revised 18 item scale (Karlsson et al., 2000)	Improved body composition; enhanced body perception compared to baseline; reduced fears and negative emotions; positive effects on well-being
Shetty et al. (2019); *N* = 60	Children aged 10–15 years; BMI > 25; Karad	Aerobic dance; control group included	6 weeks; five times per week; 45 min per session	Experimental study; pre-post test design	Posture questionnaire	Significant improvement in physical self-perception; decreased body dissatisfaction; reduction in body image dissatisfaction; increased self-confidence
Connection and connectedness						
Carraça et al. (2012); *N* = 184	Women aged 25–50 years; BMI 25–40 kg/m^2^; Portugal	Dance combined with relaxation sessions; control group included	1-year intervention and 2-year follow-up (without intervention); once per week	Randomized, controlled long-term study	Self-monitoring diary; figure rating scale (Foster et al., 1997); Body shape questionnaire (Cooper et al., 1987); social physique anxiety scale (SPAS) (Hart et al., 1989); lifestyle-PA-index (Silva et al., 2010)	Reduction in body dissatisfaction; improved body image can facilitate weight loss; enhancements across body image dimensions; increased self-awareness through physical activity; increased self-efficacy; more positive body perceptions; enhanced psychological well-being
Flow and mindfulness						
Allet et al. (2017); *N* = 54	Men and women; intervention group age 46.91 ± 10.15 and control group age 50.39 ± 8.54; BMI ≥ 30 kg/m^2^; Geneva,	Dance therapy; control group received patient education program	16-week intervention; once per week	Single-blind, quasi-randomized controlled trial	Impact of weight on quality of life-lite (IWQOL-lite)	Improved quality of life; enhanced self-worth; participants experienced freedom of movement and a renewed perception of their body
Malkina-Pykh (2012); *N* = 104	Men and women; Ø 37.6 years; BMI distribution: 31% BMI = 27.8 ± 1.3 kg/m^2^, 29% BMI = 32.7 ± 1.5 kg/m^2^, 21% BMI = 37.6 ± 1.5 kg/m^2^, 19% BMI = 44.5 ± 2.7 kg/m^2^; St. Petersburg, Russia	Rhythmic movement therapy; control group received weight management program	24 sessions, every 2 weeks; 55–65 min per session	Controlled, randomized study; pre-post-test design	Toronto Alexithymia scale (TAS-26) (Taylor et al., 1985); body image scale; multidimensional perfectionism scale (Frost et al., 1990; Hewitt et al., 1995)	Association between emotional eating and body image dissatisfaction; improvement in body image disturbances
Muller-Pinget et al. (2012); *N* = 18	Women and men; Ø 44 ± 2.4 years; BMI 36.7 ± 1.2 kg/m^2^; Switzerland	Dance therapy; no control group	36 weeks; 2 h per week	Not reported	Impact of weight on quality of life (IWQOL)-lite (Kolotkin and Crosby, 2002); Laban’s method for self-perception of walk and posture (Warner and Andrews, 1999); development movement patterns from Cohen (Payne, 2009)	Improved body awareness and mental representation; experiences of relaxation; enhanced health-related quality of life and body awareness; clearer body perception; strengthened self-image; expression of more positive emotions; increased sense of belonging; improvements in body image and psychosocial aspects of personality.
Muller-Pinget et al. (2018); *N* = 46	Women; Ø 46.91 ± 10.15 years; BMI 38.4 ± 6.6 kg/m^2^; Switzerland	Dance therapy; no control group	16 weeks; 2 h per week	No information provided	Impact of weight on quality of life (IWQOL)-lite (Kolotkin and Crosby, 2002); Laban’s method for self-perception of walk and posture (Warner and Andrews, 1999); developmental movement patterns from Cohen (Payne, 2009)	Improvement in body awareness and body perception; improved posture; increased body esteem; participants engage with mental and physical sensations; strengthened self-esteem; enhanced perception of the body as a subject independent of weight loss.
Muller-Pinget et al. (2019); *N* = 11	Women and men; Ø 43.4 ± 9 years; BMI 34.8 ± 7 kg/m^2^; Switzerland	Dance therapy; no control group	18 weeks; 90 min per week	No information provided	Rosenberg self-esteem scale (RSES) (Rosenberg et al., 1995); assessment of physical self-perception using a questionnaire (Ninot et al., 2000) “physical self-inventory” (Kolotkin and Crosby, 2002); self-questionnaire on size	Improvement in overall quality of life; increase to very high self-esteem; participants reported greater pride, self-respect, satisfaction, and self-confidence; reduction of depressive symptoms; improvement in body perception and bodily awareness.
Emotion and fantasy						
Meekums et al. (2012); *N* = 79	Women; aged 20–35 years; BMI ≥ 28; Latvia	Dance therapy; one control group without movement intervention and one with movement intervention	5-week intervention; twice per week; 1.5 h	Partially randomized controlled study; pre-post-test design	The situational inventory of body image Dysphoria (SIBID) (Cash, 2000); Rosenberg Self-Esteem Scale (Rosenberg et al., 1995)	Increased well-being and reduction of psychological symptoms (depression, anxiety, and trauma-related symptoms); improved self-esteem; more positive appearance evaluation; reduced feelings of shame; increased willingness to visit public spaces; overall positive effects on body image, self-esteem, and self-concept.

**TABLE 3:** Detailed intervention content of the included studies.

Author, year	Type of dance	Theoretical basis/conceptual framework/content	Instructor	Type of participation
Group cohesion and culture				
Azevedo et al. (2013)	Mexican folklore	No explicit theoretical framework reported; stretching exercises, free dance, and learning choreographies	School teachers	Voluntary participation with parental consent
Aesthetic fitness and technique				
Akyilmaz et al. (2023)	Zumba	No explicit theoretical framework reported; online Zumba videos categorized into beginner, intermediate, and advanced levels; participants progressed gradually to reach target heart rate over the 8-week intervention	Online via video material	Voluntary participation; recruitment following nutritional counseling and weight reduction therapy
Shetty et al. (2019)	Aerobic dance	No explicit theoretical framework reported; warm-up exercises, aerobic dance sequences, and cool-down exercises	Not specified	Voluntary participation
Connection and conectedness				
Carraça et al. (2012)	Dance combined with relaxation exercises	Intervention based on the principles of Self-Determination Theory (Deci and Ryan, 1985); focus on movement regulation and weight control; based on the work of Cooper et al. (2003)	Not specified	Voluntary participation; recruitment via internet and media announcements
Flow and mindfulness				
Allet et al. (2017)	Dance therapy	Theoretical foundations of dance therapy; free and creative dance instruction. Each session begins with a warm-up, followed by assessment of muscular and psychological tension, relaxation, and breathing exercises, then 60 min of dance. Participants observe their feelings during movement to build a relationship with their personal space and develop a mental representation of their body image.	Dance therapist	Voluntary participation; participants interested in dance
Malkina-Pykh (2012)	Rhythmic movement therapy	Based on the principles of rhythmic movement therapy (RMT); structured in three phases: preliminary assessment, Phase 1 (CBT-B), and Phase 2 (RMT treatment); each session addresses a core problem; the final RMT phase ends with reflection and discussion; personality is assessed through diagnostic exercises; emotional expression is promoted through resource exercises; individual improvements in body image, interpersonal relationships, and quality of thinking are analyzed using test exercises.	Psychologists trained for 1 year in cognitive-behavioral therapy and rhythmic movement therapy for eating disorders and obesity	Referred by specialists from a weight management program and randomly selected from there
Muller-Pinget et al. (2012)	Dance therapy	Principles, concepts, and techniques of authentic dance and movement training within a dance therapy approach. Session structure: warm-up with fast movements to achieve a state of mobility and enhance bodily sensation (Laban, 1971); second phase addressing muscular and physical tension through breathing and relaxation exercises, with movement retraining based on sensorimotor relationships and phylogenetic and ontogenetic movement history; use of authentic dance techniques (Adler, 2002) to explore sensations, emotions, memories, and mental representations.	Not specified	Voluntary participation
Muller-Pinget et al. (2018)	Dance therapy	Principles and concepts of dance therapy (five dimensions of therapeutic education): cognitive, affective-emotional, subcognitive, metacognitive, and perceptive dimensions; observation of tension states; exploration of body language; improvisational dance based on individual experience; work on self-perception goals.	Not specified	Voluntary participation
Muller-Pinget et al. (2019)	Dance therapy	Principles and concepts of dance therapy; choreography; improvisation; development of body awareness in motion and at rest to explore emotions and thoughts; perception and release of physical and mental tension; explorations of links between bodily and emotional experience and their expression through dance therapy	Not specified	Voluntary participation
Emotion and fantasy				
Meekums et al. (2012)	Dance therapy	Theoretical foundations based on Meekums’ emphasis on movement metaphors and the WISE/EMPoweR model for creative change, including phases of warm-up, creative activities, insight, and evaluation within a therapeutic relationship (Meekums, 2002; Payne, 2013)	Study authors (M.Sc. in Art Therapy)	Voluntary participation

The component-based categorization allows for (1) conceptual comparability despite methodological differences, (2) a theory-driven analysis of potential mechanisms affecting body image and self-worth, (3) differentiation according to technical, emotional, or social intervention emphases, and (4) identification of patterns and differences across interventions. This transparent classification enhances the reproducibility and interpretability of the synthesis.

### 2.6 Assessment of methodological quality of included studies

The methodological quality of the included studies was evaluated according to study design.

For qualitative studies and the qualitative components of mixed-method studies, the Joanna Briggs Institute (JBI) “Checklist for Qualitative Research”^2^ (Lockwood et al., 2015) was applied. Evaluation was based on ten criteria, using the response categories: yes, no, unclear, or not applicable. The results are presented in Table 4.

**TABLE 4:** Quality of studies with qualitative data collection methods (1 qualitative, 3 mixed-methods).

Author	Q1	Q2	Q3	Q4	Q5	Q6	Q7	Q8	Q9	Q10
Group cohesion and culture										
Azevedo et al. (2013)	Yes	Yes	Yes	Yes	Yes	Yes	No	Yes	Unclear	Yes
Aesthetics fitness and technique										
Akyilmaz et al. (2023)	Yes	Yes	Yes	Yes	Yes	Unclear	No	Unclear	Yes	Yes
Connection and connectedness										
Carraça et al. (2012)	Yes	Yes	Yes	Yes	Yes	No	No	Unclear	Yes	Yes
Flow and mindfulness										
Muller-Pinget et al. (2012)	Yes	Yes	Yes	Yes	Yes	Yes	No	Unclear	Unclear	Yes

Q1: Is there congruity between the stated philosophical perspective and the research methodology? Q2: Is there congruity between the research methodology and the research question or objectives? Q3: Is there congruity between the research methodology and the methods used to collect data? Q4: Is there congruity between the research methodology and the representation and analysis of data? Q5: Is there congruity between the research methodology and the interpretation of results? Q6: Is there a statement locating the researcher culturally or theoretically? Q7: Is the influence of the researcher on the research, and vice-versa, addressed? Q8: Are the participants, and their voices, adequately represented? Q9: Is the research ethical according to current criteria or, for recent studies, and is there evidence of ethical approval by an appropriate body? Q10: Do the conclusions drawn in the research report flow from the analysis, or interpretation, of the data?

For quantitative studies and the quantitative components of mixed-method studies, the JBI “Checklist for Randomized Studies”^3^ (Barker et al., 2023) was used. The 13 criteria were rated using the values 1, 0, –1, or not applicable. Detailed results are summarized in Table 5. Additionally, Table 6 presents the mean scores graphically using arrow symbols to provide a clear comparison of methodological quality. Table 6 also highlights key quality indicators such as authorship, sample sizes, effect sizes, randomization procedures, and the presence of control groups.

**TABLE 5:** Quality of studies using quantitative data collection methods (6 quantitative, 3 mixed-methods).

Author	Q1	Q2	Q3	Q4	Q5	Q6	Q7	Q8	Q9	Q10	Q11	Q12	Q13
Aesthetic fitness and technique													
Akyilmaz et al. (2023)	1	0	1	0	0	0	0	1	1	1	1	1	1
Shetty et al. (2019)	1	0	1	0	0	1	0	1	1	0	1	1	1
Connection and connectedness													
Carraça et al. (2012)	1	0	1	–1	1	1	1	1	1	1	1	1	1
Flow and mindfulness													
Allet et al. (2017)	1	1	1	1	1	1	–1	1	1	1	1	1	1
Malkina-Pykh (2012)	1	0	1	–1	–1	1	–1	1	1	0	1	1	1
Muller-Pinget et al. (2012)	0	–1	1	–1	–1	1	–1	1	1	0	1	1	–1
Muller-Pinget et al. (2018)	1	1	1	1	–1	1	1	1	1	1	1	1	1
Muller-Pinget et al. (2019)	0	–1	1	–1	–1	1	–1	1	1	0	1	1	1
Emotion and fantasy													
Meekums et al. (2012)	–1	0	1	–1	–1	1	–1	1	1	0	1	1	1

The scale values range from “Yes, present” (1) to “No, not present” (0) and “Unclear” (-1). Q1: Was true randomization used for assignment of participants to treatment groups? Q2: Was allocation to treatment groups concealed? Q3: Were treatment groups similar at the baseline? Q4: Were participants blind to treatment assignment? Q5: Were those delivering the treatment blind to treatment assignment? Q6: Were treatment groups treated identically other than the intervention of interest? Q7: Were outcome assessors blind to treatment assignment? Q8: Were outcomes measured in the same way for treatment groups? Q9: Were outcomes measured in a reliable way? Q10: Was follow up complete and if not, were differences between groups in terms of their follow up adequately described and analyzed? Q11: Were participants analyzed in the groups to which they were randomized? Q12: Was appropriate statistical analysis used? Q13: Was the trial design appropriate and any deviations from the standard RCT design (individual randomization, parallel groups) accounted for in the conduct and analysis of the trial?

**TABLE 6:** Summary of study quality using quantitative methods (1 quantitative, 3 mixed-methods).

Author	Sample size	Control group?	Randomized no.	Effect size?	M overall quality	Overall quality
Aesthetics fitness and technique						
Akyilmaz et al. (2023)	150	Yes	Yes	Yes	0.62	↑
Shetty et al. (2019)	60	Yes	Yes	No	0.62	↑
Connection and connectedness						
Carraça et al. (2012)	184	Yes	Yes	Yes	0.46	↑
Flow and mindfulness						
Allet et al. (2017)	54	Yes	Yes	Yes	0.85	↑
Malkina-Pykh (2012)	104	Yes	Yes	Yes	0.38	↑
Muller-Pinget et al. (2012)	18	No	No	No	0.08	→
Muller-Pinget et al. (2018)	46	Yes	Yes	No	0.92	↑
Muller-Pinget et al. (2019)	11	No	No	No	0.31	↑
Emotion and fantasy						
Meekums et al. (2012)	79	Yes	Partially-randomized	No	0.23	→

↑, Mean in the upper third of the quality rating scale (0.33 < mean < 1); →, Mean in the middle third of the rating scale (–0.33 < mean ≤ 0.33); ↓, Mean in the lower third of the rating scale (–1 < mean ≤ – 0.33).

No studies were excluded based on methodological quality, primarily due to the overall limited evidence base on dance interventions for individuals with obesity. The assessment of methodological quality was therefore considered in the presentation and discussion of results. The results of the quality assessment were explicitly taken into account when interpreting the study findings. Studies with lower methodological quality (e.g., unclear randomization procedures, small sample sizes, or absence of control groups) were interpreted with greater caution regarding the strength of their evidence. In contrast, methodologically more robust studies were given greater weight in deriving conclusion about the potential effects of dance-based interventions. Overall, the strength of the aggregated findings is therefore considered limited in light of the heterogeneity in study quality. The assessment of methodological quality is thus directly integrated into both the results presentation and the discussion.

**FIGURE 2:**
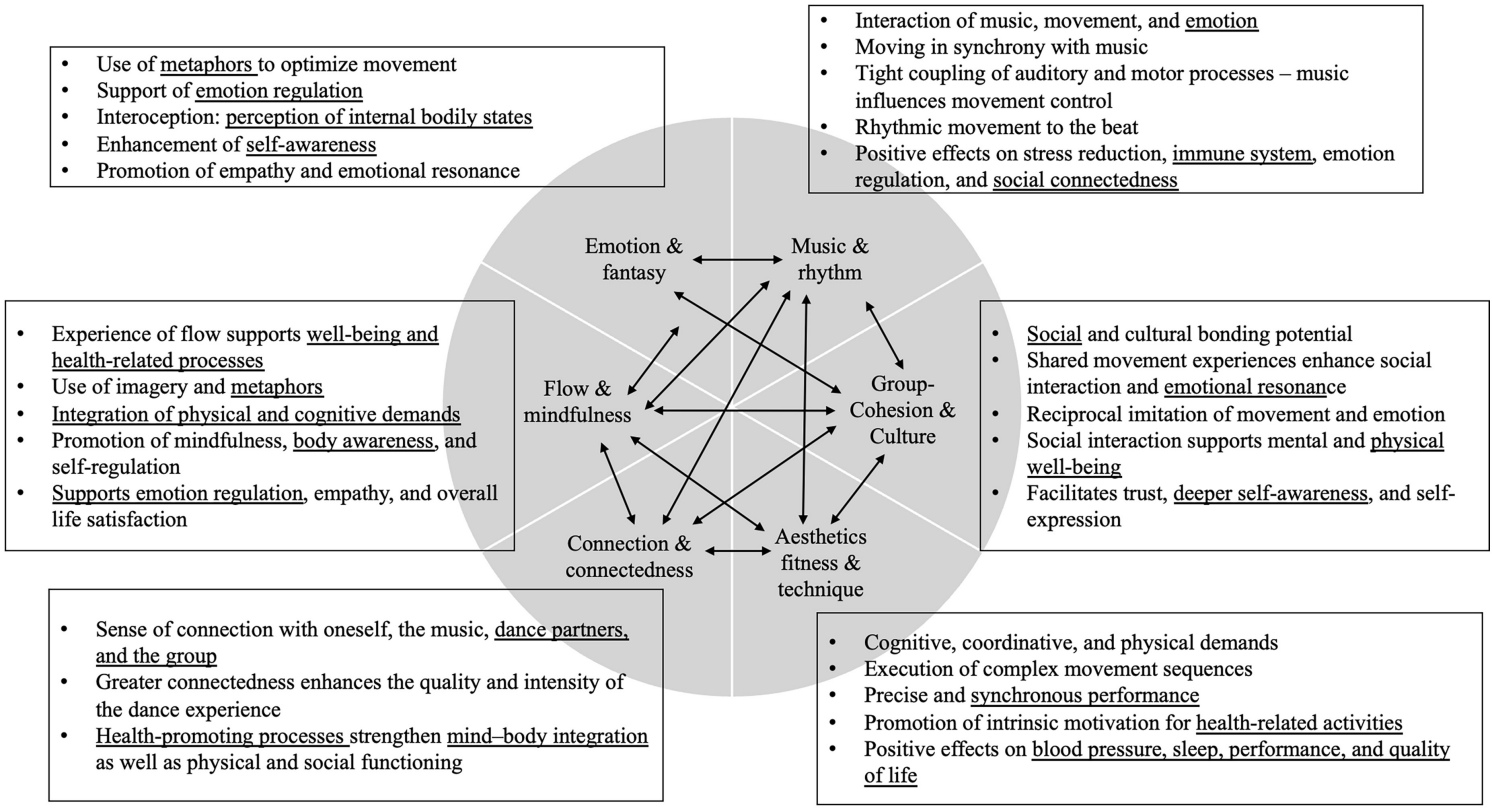
Content overlaps within the Wheel of Dance according to Christensen et al. (2021) (own illustration).

The assignment of studies to the components of the *Wheel of Dance* was based on the described intervention content and objectives. However, clear categorization was not possible in all cases, as the categories defined by Christensen et al. (2021) show conceptual overlaps (see Figure 2).

The classification procedure can be exemplified using the study by Shetty et al. (2019): The intervention is primarily assigned to the category *Aesthetic Fitness and Technique*, as its focus is on physical training, coordination, and structured movement sequences. The structure, consisting of a warm-up, an aerobic dance phase, and a cool-down, reflects a classical fitness-oriented training format. Other categories are only marginally addressed: *Music and Rhythm* plays a functional role, as aerobic dance movements are typically music-bound; *Group Cohesion and Culture* may be implicitly engaged through group training and culturally influenced movement patterns but are not central to the intervention. The categories *Connection and Connectedness, Flow and mindfulness*, and *Emotion and Fantasy* are not explicitly targeted, as the focus is primarily on physical activity and health-related behavior, although certain aspects may be indirectly involved.

Fundamentally, the individual components emphasize different analytical aspects: *Music and Rhythm* highlights musical-rhythmic structures, *Group Cohesion and Culture* emphasizes social and cultural dynamics, *Aesthetic Fitness and Technique* focuses on physical and technical demands, *Connection and connectedness* captures subjectively experienced connectedness, *Flow and mindfulness* centers on present-focused attention, and *Emotion and Fantasy* involves the use of metaphors and imagery.

**FIGURE 3:**
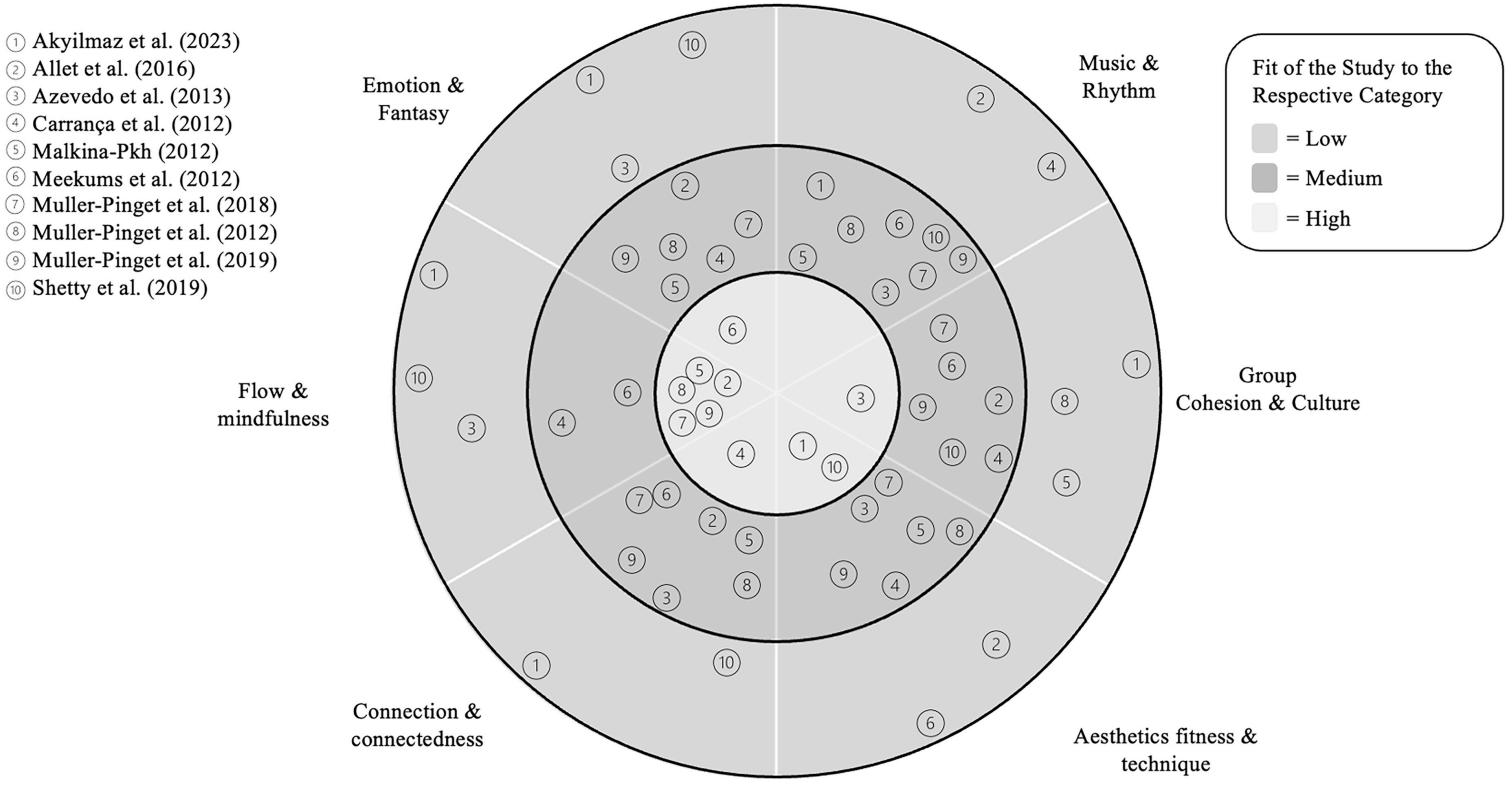
Direct and indirect assignment of studies according to the categories of the Wheel of Dance by Christensen et al. (2021) (own illustration).

The complete classification of all included studies is illustrated in Figure 3.

## 3 Results

**FIGURE 4:**
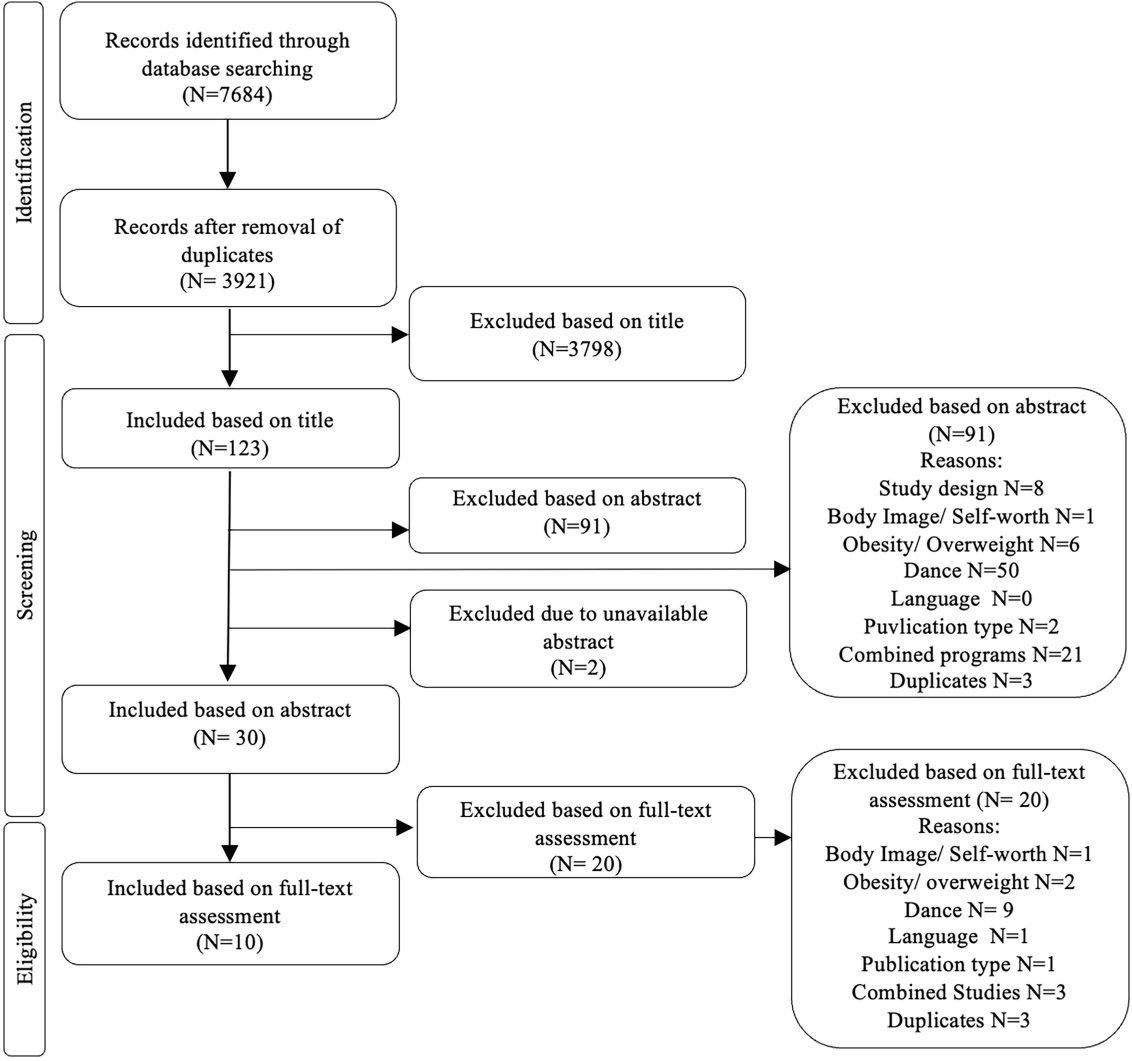
Flowchart of the search and study selection process.

Figure 4 illustrates the flow of the systematic literature search in accordance with the PRISMA guidelines, including the number of identified records as well as inclusions and exclusions at each stage of the selection process. The reasons for exclusion are transparently reported for each phase. The initial database search yielded 7.684 records. After removing duplicates and conducting title and abstract screening based on the predefined inclusion and exclusion criteria, 30 articles were assessed for full-text eligibility. Ten studies met all inclusion criteria and were included in the analysis.

Nine of the ten included studies were conducted in European countries, with one additional study from the United States. All studies were published after 2010. Overall, the included studies differed regarding intervention approaches, intervention durations, research designs, and reported outcomes.

Given this heterogeneity and the assumption that intervention effects may vary depending on specific program characteristics, the framework of Christensen et al. (2021) was used as an analytical structure. The Wheel of Dance model provides a systematic approach for classifying interventions and improves comparability across findings.

Most studies could be assigned to the components of the Wheel of Dance: one study to *Group Cohesion and Culture* (Azevedo et al., 2013), two studies to *Aesthetic Fitness and technique* (Akyilmaz et al., 2023; Shetty et al., 2019), one study to *Connection and connectedness* (Carraça et al., 2012), five studies to *Flow and Mindfulness* (Allet et al., 2017; Malkina-Pykh, 2012; Muller-Pinget et al., 2012, 2018, 2019), and one study to *Emotion and Fantasy* (Meekums et al., 2012). No study was exclusively assigned to the Music and Rhythm component.

### 3.1 Participants

The studies investigated different target populations in terms of age, sex, and BMI. Two studies focused on children and adolescents aged 8–15 years with BMI values above 25 kg/m^2^ (Azevedo et al., 2013; Shetty et al., 2019). Four studies included only adult women aged between 25 and 57 years with BMI values ranging from 25 to 45 kg/m^2^ (Akyilmaz et al., 2023; Carraça et al., 2012; Meekums et al., 2012; Muller-Pinget et al., 2018). Four additional studies involved mixed samples of women and men aged 30–59 years with BMI values between 26 and 47 kg/m^2^ (Allet et al., 2017; Malkina-Pykh, 2012).

The sample sizes vary considerably across studies. Two studies included fewer than 20 participants (11 and 18 individuals) (Muller-Pinget et al., 2012, 2019), five studies used sample sizes between 43 and 79 participants (Allet et al., 2017; Azevedo et al., 2013; Meekums et al., 2012; Muller-Pinget et al., 2018; Shetty et al., 2019), and three studies included 104–184 participants (Akyilmaz et al., 2023; Carraça et al., 2012; Malkina-Pykh, 2012).

### 3.2 Setting

Of the ten included studies, three were conducted in clinical settings (inpatient or outpatient care) (Allet et al., 2017; Malkina-Pykh, 2012; Muller-Pinget et al., 2019). Two studies involving children aged 8–15 years were carried out in school settings (Azevedo et al., 2013; Shetty et al., 2019). One study was conducted online in a home-based environment (Akyilmaz et al., 2023), one at a university setting (Meekums et al., 2012), and one in an art-therapeutic institution (Foundation ART-Therapie) (Muller-Pinget et al., 2018). In two studies, the implementation site was not reported (Carraça et al., 2012; Muller-Pinget et al., 2012).

### 3.3 Research design and approach

The included studies demonstrate methodological diversity and encompass qualitative, quantitative, and mixed-method approaches, ranging from ethnographic and interview-based investigations to controlled experimental and quasi-randomized study designs.

One study used a randomized design (Muller-Pinget et al., 2018), one employed an experimental design (Shetty et al., 2019), and six used a mixed-method design (Akyilmaz et al., 2023; Allet et al., 2017; Azevedo et al., 2013; Carraça et al., 2012; Malkina-Pykh, 2012; Meekums et al., 2012). Two studies did not provide detailed information regarding the study design (Muller-Pinget et al., 2012, 2019). Six studies included a control group (Akyilmaz et al., 2023; Allet et al., 2017; Carraça et al., 2012; Malkina-Pykh, 2012; Meekums et al., 2012; Shetty et al., 2019).

Most studies employed at least one questionnaire assessing body image and self-worth (Akyilmaz et al., 2023; Allet et al., 2017; Carraça et al., 2012; Malkina-Pykh, 2012; Meekums et al., 2012; Muller-Pinget et al., 2012, 2018, 2019; Shetty et al., 2019). One study combined questionnaires with a self-observation diary (Carraça et al., 2012), and one study relied exclusively on qualitative interviews (Azevedo et al., 2013). Detailed data collection methods are summarized in Table 2.

Intervention duration varied considerably. Four studies lasted more than 20 weeks (Azevedo et al., 2013; Carraça et al., 2012; Malkina-Pykh, 2012; Muller-Pinget et al., 2012), while six studies lasted < 20 weeks (Akyilmaz et al., 2023; Allet et al., 2017; Meekums et al., 2012; Muller-Pinget et al., 2018, 2019; Shetty et al., 2019). The frequency of dance sessions ranged from once per week (Allet et al., 2017; Azevedo et al., 2013; Carraça et al., 2012; Malkina-Pykh, 2012; Muller-Pinget et al., 2019) to five times per week (Akyilmaz et al., 2023; Meekums et al., 2012; Muller-Pinget et al., 2012, 2018; Shetty et al., 2019).

Sample sizes ranged from 11 (Muller-Pinget et al., 2019) to 184 participants (Carraça et al., 2012). Eight studies included more than 20 participants (Akyilmaz et al., 2023; Allet et al., 2017; Azevedo et al., 2013; Carraça et al., 2012; Malkina-Pykh, 2012; Meekums et al., 2012; Muller-Pinget et al., 2018; Shetty et al., 2019), and six of these studies included more than 50 participants (Akyilmaz et al., 2023; Allet et al., 2017; Carraça et al., 2012; Malkina-Pykh, 2012; Meekums et al., 2012; Muller-Pinget et al., 2018; Shetty et al., 2019). Only two studies reported sample sizes below 20 participants (Muller-Pinget et al., 2012, 2019).

Eight studies specified the dance style applied. Five used dance therapy approaches (Allet et al., 2017; Meekums et al., 2012; Muller-Pinget et al., 2012, 2018, 2019), one used rhythmic movement therapy (Malkina-Pykh, 2012), one used Mexican folklore dance (Azevedo et al., 2013), one used Zumba (Akyilmaz et al., 2023), and one used aerobic dance (Shetty et al., 2019). The intervention content of Carraça et al. (2012) was not specified in detail.

### 3.4 Study quality

The methodological quality of the included studies was evaluated to assess the robustness and interpretability of the reported findings. Tables 4–6 provide an overview of the fulfillment of the respective quality criteria across studies. One study employed an exclusively qualitative design (Azevedo et al., 2013), six studies used purely quantitative approaches (Allet et al., 2017; Malkina-Pykh, 2012; Meekums et al., 2012; Muller-Pinget et al., 2018, 2019; Shetty et al., 2019), and three followed a mixed-methods design (Akyilmaz et al., 2023; Carraça et al., 2012).

Regarding qualitative studies and qualitative components of mixed-methods studies, one study met 80% of the quality criteria (Azevedo et al., 2013), while the remaining three achieved approximately 70% each (Akyilmaz et al., 2023; Carraça et al., 2012; Muller-Pinget et al., 2012). All four studies demonstrated clear methodological coherence and derived conclusions consistently from analysis and interpretation. Two studies additionally reflected their cultural and theoretical positioning (Azevedo et al., 2013; Muller-Pinget et al., 2019). None explicitly addressed potential reciprocal influence between researchers and the research process. Participant perspectives were directly represented through interview quotations in one study (Azevedo et al., 2013), and two studies explicitly reported adherence to ethical research standards (Akyilmaz et al., 2023; Carraça et al., 2012).

Nine studies included quantitative designs or quantitative components (Akyilmaz et al., 2023; Allet et al., 2017; Carraça et al., 2012; Malkina-Pykh, 2012; Meekums et al., 2012; Muller-Pinget et al., 2012, 2018, 2019; Shetty et al., 2019). Seven studies demonstrated high methodological quality (Akyilmaz et al., 2023; Allet et al., 2017; Carraça et al., 2012; Malkina-Pykh, 2012; Muller-Pinget et al., 2018, 2019; Shetty et al., 2019), while two achieved moderate quality (Meekums et al., 2012; Muller-Pinget et al., 2012). Limitations were mainly related to the lack of blinding of participants and researchers. Two studies did not include a control group (Muller-Pinget et al., 2012, 2019), two did not apply randomization (Muller-Pinget et al., 2012, 2019), and one used partial randomization (Meekums et al., 2012). Five studies did not report effect sizes (Meekums et al., 2012; Muller-Pinget et al., 2012, 2018, 2019; Shetty et al., 2019). Overall, most studies met core quality criteria associated with randomized controlled designs.

### 3.5 Intervention content and their effects on body image and self-worth

Due to heterogeneity in study design, intervention characteristics, and outcome measures, findings were synthesized narratively. Accordingly, a meta-analysis or purely method-based synthesis was not possible.

Moreover, the included studies exhibited heterogeneity in the terminology used to describe the core constructs of self-worth and body image. Table 7 illustrates that different terms were used, some of which were applied synonymously despite being theoretically attributable to distinct constructs. Different terms were used to describe body image and self-worth-related constructs across studies.

**TABLE 7:** Synonymously used and thematically related terms for body image and self-worth.

Body image	Self-worth
Body perception, body experience, self-image, physical self-perception, body awareness, bodily identity, body representation, body feeling, self-image, body-related self-concept, bodily self-view, bodily self-perception, body concept, bodily self-assessment, body awareness, body perspective, body representation	Self-esteem, self-respect, self-confidence, self-worth, self-regard, self-esteem evaluation, self-image, self-assessment, self-acceptance, self-respect, self-perception, self-efficacy, self-evaluation, self-worth appreciation, identity, personality

Overall, nine studies, including quantitative and mixed-methods studies, examine different aspects of body image. While all nine investigate the overarching construct of body image (Akyilmaz et al., 2023; Allet et al., 2017; Carraça et al., 2012; Malkina-Pykh, 2012; Meekums et al., 2012; Muller-Pinget et al., 2012, 2018, 2019; Shetty et al., 2019), the specific body image components addressed vary considerably.

Six studies focus on body satisfaction (Akyilmaz et al., 2023; Carraça et al., 2012; Malkina-Pykh, 2012; Muller-Pinget et al., 2018, 2019; Shetty et al., 2019), and eight studies address body perception (Akyilmaz et al., 2023; Carraça et al., 2012; Malkina-Pykh, 2012; Meekums et al., 2012; Muller-Pinget et al., 2012, 2018, 2019; Shetty et al., 2019). In addition, individual studies examined evaluative components of body image (one of nine) (Carraça et al., 2012), dysfunctional aspects (two of nine) (Carraça et al., 2012; Malkina-Pykh, 2012), or obesity-related body image disturbances (two of nine) (Malkina-Pykh, 2012; Meekums et al., 2012).

Self-worth was also examined in nine studies. Eight studies investigated global self-worth (Allet et al., 2017; Azevedo et al., 2013; Carraça et al., 2012; Malkina-Pykh, 2012; Muller-Pinget et al., 2012, 2018, 2019). In addition, specific facets were addressed, including self-efficacy (one of nine) (Carraça et al., 2012), self-conscious (four of nine) (Azevedo et al., 2013; Carraça et al., 2012; Muller-Pinget et al., 2018, 2019), self-confidence (four of nine) (Azevedo et al., 2013; Muller-Pinget et al., 2012, 2018; Shetty et al., 2019), self-empowerment (one of nine) (Azevedo et al., 2013), and self-acceptance (two of nine) (Muller-Pinget et al., 2018, 2019).

The relationship between body image and self-worth was analyzed in four studies; three of these focused on the direct association between the two constructs (Carraça et al., 2012; Meekums et al., 2012; Shetty et al., 2019). Furthermore, three studies examined the linkage between body image, self-worth, and body weight (Carraça et al., 2012; Meekums et al., 2012; Muller-Pinget et al., 2012).

#### 3.5.1 Group cohesion and culture

The study by Azevedo et al. (2013) is the only included study whose dance intervention can be assigned to the “Group Cohesion and Culture” component of the Wheel of Dance. The intervention centered on Mexican folkloric dance, integrating cultural identity, communal learning, and intergenerational knowledge transmission. Over a period of 2 years, girls aged 7–11 participate in instructor-led dance sessions five times per week, each lasting between 1.5 and 3 h. The program included stretching exercises, free movement to music, and the rehearsal of choreographies performed in traditional attire. No explicit theoretical framework was reported. An additional objective was the reduction of daily screen time in favor of culturally embedded, community-based movement practice.

The findings indicate pronounced positive effects on both self-worth and body image. Parents reported increased self-confidence, enhanced global self-esteem, more positive self-perception, improved academic performance, and changes in body weight. Moreover, the girls demonstrate greater confidence in social interactions and increased autonomy in everyday life. Particularly emphasized was the development of a stronger awareness of their cultural identity, a heightened sense of belonging, and the formation of new social relationships. Younger participants benefit from learning alongside older girls, which appears to strengthen their social integration and sense of competence. Parents reported increased self-confidence, self-esteem, positive self-perception, and stronger social participation among participants.

#### 3.5.2 Aesthetic fitness and technique

In the study by Shetty et al. (2019), schoolchildren participate in a daily 45-min aerobic dance program over a period of 6 weeks. Sessions include warm-up exercises, standardized aerobic steps (e.g., jumping jacks, V-step, and jumps), and a cool-down phase. In addition, participants aged 10–15 years receive nutritional counseling.

Clinically relevant reductions in body weight, fat mass, and waist-to-hip ratio are observed following the intervention. At the same time, participants reported improvements in body image and a more differentiated bodily self-perception. Increases in self-confidence, self-assurance, and overall physical fitness were also observed.

The intervention investigated by Akyilmaz et al. (2023) consists of an 8-week Zumba program conducted five times per week by women aged 20–40 years using online videos in a home-based setting. Training intensity was progressively increased until participants reached their individual target heart rate. Significant physiological changes are observed, including reductions in body weight, fat mass, body fat percentage, and waist-to-hip ratio.

Notably, significant effects on body image are reported: participants described improved body perception and a more positive overall body image, whereas scores in the walking control group deteriorate. The findings suggest that dance-based exercise formats may positively affect both evaluative and perceptual components of body image. In addition, a reduction in psychological stress is observed, indicating the potentially holistic effectiveness of the Zumba training.

#### 3.5.3 Connection and connectedness

The study by Carraça et al. (2012) can be assigned to the “Connection and connectedness” component of the *Wheel of Dance*, as it examines social, emotional, and self-regulatory processes within a dance-based intervention. The program is conducted with women aged 25–50 years and extends over a 12-month period, comprising weekly dance and relaxation sessions; follow-up assessments are conducted after 24 months. The study was theoretically grounded in Self-Determination Theory (Deci and Ryan, 1985) and in concepts of self-regulated weight control (Cooper et al., 2003). The intervention aims to promote physical activity, strengthen the evaluative component of body image (i.e., body acceptance and satisfaction), and reduce dysfunctional preoccupation with physical appearance.

The findings indicate marked improvements in body image. Although effects are observed in both the intervention and control groups, positive changes are more pronounced in the intervention group, which is associated with higher levels of physical activity. At baseline, both groups exhibit substantially impaired body image, and even after 12 months no full normalization is achieved. Nevertheless, the intervention group demonstrates a greater reduction in dysfunctional investment, defined as the excessive importance attributed to physical appearance for self-worth and behavior, which is accompanied by improvements in self-regulation.

After 12 months, both dimensions of body image improve significantly; however, at the 24-month follow-up, slight increases in body dissatisfaction and dysfunctional investment are observed. At follow-up, participants showed changes in body image dimensions and dysfunctional investment in appearance. Overall, participants report attributing less importance to physical appearance for their self-worth and psychological well-being. These results underscore the role of social integration and emotional connectedness within the context of the dance-based intervention.

#### 3.5.4 Flow and mindfulness

Five studies can be assigned to this component (Allet et al., 2017; Malkina-Pykh, 2012; Muller-Pinget et al., 2012, 2018, 2019). Four are based on dance therapy approaches (Allet et al., 2017; Muller-Pinget et al., 2012, 2018, 2019), and one on rhythmic movement therapy (Malkina-Pykh, 2012). The interventions included elements such as free movement, improvisation, breathing exercises, relaxation, and body awareness tasks. Allet et al. (2017) conduct a 16-week dance therapy intervention with male and female participants (Ø 46.91 ± 10.15 years). Free and creative movement sequences, combined with breathing and relaxation exercises, emphasized the conscious perception of emotions, bodily sensations, and mental body representations. Muller-Pinget et al. (2012) work with a mixed-gender group (Ø 44 ± 2.4 years) over 36 weeks, meeting twice weekly. The intervention is based on Authentic Movement as described by Adler (2002) and Laban (1971), with a focus on enhancing body perception and increasing sensitivity to muscular tension. The study by Muller-Pinget et al. (2018) applies a five-component therapeutic educational model encompassing cognitive, affective-emotional, subcognitive, metacognitive, and perceptual dimensions. The intervention emphasizes self-perception, body language, improvisation, as well as affective and subcognitive processes. Participants (Ø 46.91 ± 10.15 years) attend weekly 2 h sessions over a period of 16 weeks. In Muller-Pinget et al. (2019), choreographic work is combined with improvisation to enhance awareness of physical and emotional strain. The mixed-gender group (Ø 43.4 ± 9 years) participates in weekly 90-min sessions across 18 weeks. The rhythmic movement therapy described by Malkina-Pykh (2012) comprises 24 sessions conducted biweekly with a mixed-gender sample (Ø 37.6 years). The intervention integrates diagnostic, resource-oriented, and body-analytical exercises within a three-phase structure (pre-assessment, behavioral therapy, and rhythmic movement therapy). The focus is placed on body image, emotional expressivity, and cognitive processes.

The findings indicate that all dance and movement therapy interventions lead to improvements in body image, body perception, and participants’ emotional experiences. Malkina-Pykh (2012) demonstrates that embodied, somatic experiences are central to therapeutic change, whereas traditional talk-based therapy alone shows comparatively limited effects.

Several studies report improvements in body image following dance-based interventions (Allet et al., 2017; Malkina-Pykh, 2012; Muller-Pinget et al., 2012). Particularly notable is the description of participants in Malkina-Pykh (2012): at the beginning of the intervention, they demonstrate limited access to proprioceptive and kinesthetic perception, with their focus primarily directed toward visual and emotional aspects. Participants reported changes in body perception and increased awareness of bodily sensations (Muller-Pinget et al., 2018). Over the course of the intervention, both body awareness and the mental representation of the body improve. Participants learn to establish new relationships with themselves and to perceive their bodies not as rigid mass but as fluid and dynamic (Muller-Pinget et al., 2012). Perceptions of posture also change positively (Muller-Pinget et al., 2012, 2018).

In addition, four studies report enhanced quality of life (Allet et al., 2017; Muller-Pinget et al., 2012, 2019), which is particularly attributed to processes of improvisation, the release from rigid aesthetic movement ideals, and a deeper immersion in bodily and affective experiences (Muller-Pinget et al., 2019).

Several studies reported improvements in body image despite no changes in body weight (Allet et al., 2017; Muller-Pinget et al., 2012, 2019). Nevertheless, participants perceive their weight less negatively over the course of the intervention. Allet et al. (2017) report that participants experienced a sense of freedom in movement despite their weight, which enables them to rediscover their bodies. Dancing allows them to experience sensations in new ways and to develop a transformed relationship with their bodies (Muller-Pinget et al., 2018).

Four studies also document significant improvements in self-worth (Allet et al., 2017; Muller-Pinget et al., 2012, 2018, 2019). Beginning from markedly impaired self-esteem and distorted body perception (Muller-Pinget et al., 2012), participants report increased self-confidence, pride, self-acceptance, and satisfaction (Muller-Pinget et al., 2018, 2019). In particular, the transformation of movement patterns and the embodiment of emotional expression contribute to these changes. Malkina-Pykh (2012) further demonstrates that emotional expressivity and self-perception improve substantially throughout the intervention.

Group dynamics play a central role across all studies (Allet et al., 2017; Muller-Pinget et al., 2012, 2018, 2019), fostering the experience of not being alone with one’s emotions and difficulties (Allet et al., 2017) and thereby supporting the development of a more positive self-relationship as well as improved social connectedness (Muller-Pinget et al., 2018).

#### 3.5.5 Emotion and fantasy

The study by Meekums et al. (2012) investigates the effects of dance movement therapeutic processes on body image, self-worth, and psychological well-being in women aged 20–35 years. The intervention is grounded in Meekums’ concept of movement metaphors as well as the WISE/EMPoweR model, which promotes creative change processes through the integration of movement, emotion, and imagination (Meekums, 2002; Payne, 2013). Over a period of 5 weeks, participants attend ten 90-min sessions consisting of warm-up exercises, creative movement sequences, reflective components, and the development of a therapeutic relationship. The results demonstrate pronounced positive effects, including increased general well-being, reduction of psychological symptoms, particularly depressive complaints, decreased body image disturbances, and enhanced self-worth. Participants showed improvements in body image, psychological well-being, and self-worth. No changes are observed in the control group, underscoring the specific effectiveness of the dance movement therapeutic intervention.

### 3.6 Additional findings and contextual influencing factors

Beyond the effects on body image and self-worth, the studies identify further relevant aspects. Body weight emerges as a central factor: nine of the ten studies address its change and relevance during the intervention (Akyilmaz et al., 2023; Allet et al., 2017; Azevedo et al., 2013; Carraça et al., 2012; Meekums et al., 2012; Muller-Pinget et al., 2012, 2018, 2019; Shetty et al., 2019), and four specifically analyze the relationship between body weight and body image (Akyilmaz et al., 2023; Carraça et al., 2012; Malkina-Pykh, 2012; Meekums et al., 2012).

Social interaction proves to be a key influencing factor: seven studies address social processes, with six emphasizing the relevance of shared movement experiences for perceived social integration (Allet et al., 2017; Azevedo et al., 2013; Meekums et al., 2012; Muller-Pinget et al., 2012, 2018, 2019). Four studies additionally report positive effects of social interaction on self-worth (Allet et al., 2017; Carraça et al., 2012; Meekums et al., 2012; Muller-Pinget et al., 2019), highlighting the importance of relational processes within the interventions.

## 4 Discussion

This systematic review provides a structured overview of studies examining dance-specific movement interventions for individuals with obesity and their effects on body image and self-worth. The findings indicate that dance, ranging from aesthetic-creative formats to structured exercise programs, can yield positive effects, including improvements in body perception and body satisfaction, as well as increases in self-confidence, self-awareness, self-acceptance, and overall quality of life.

At the same time, the evidence base remains limited. The small number of available studies, considerable methodological heterogeneity, variations in study design, and differences in intervention content and duration restrict comparability and limit the interpretability of the findings.

### 4.1 The effects of dance through self-awareness and social resonance

The findings of this review indicate that dance-based movement interventions for individuals with obesity can induce positive changes in body image and self-worth (Akyilmaz et al., 2023; Allet et al., 2017; Azevedo et al., 2013; Carraça et al., 2012; Malkina-Pykh, 2012; Meekums et al., 2012; Muller-Pinget et al., 2012, 2018, 2019; Shetty et al., 2019). Their effects appear to be driven less by physical activity itself than by the specific combination of embodiment, creativity, and social resonance (Schwender et al., 2025), consistent with theoretical models of embodied cognition (Koch, 2011; Kosmas and Zaphiris, 2018). The results further demonstrate that dance interventions can produce both short-term improvements (Akyilmaz et al., 2023) and more sustained long-term changes (Annesi, 2017; Carraça et al., 2012; Christensen et al., 2021), likely due to the embodied, holistic learning processes that engage multiple facets of the self simultaneously.

The included studies reported clinically meaningful improvements in both evaluative and dysfunctional dimensions of body image (Malkina-Pykh, 2012; Meekums et al., 2012; Muller-Pinget et al., 2012, 2018; Shetty et al., 2019). Dance-based interventions promoted a more differentiated body awareness, improved body perception, enhanced bodily feeling, and strengthened sensorimotor self-perception (Malkina-Pykh, 2012; Meekums et al., 2012; Muller-Pinget et al., 2012, 2018; Shetty et al., 2019), leading to an increasing experience of the body as a sensing and acting subject (Muller-Pinget et al., 2018).

Malkina-Pykh (2012) demonstrates that changes in body image are driven less by language-based cognitive processes and more by embodied somatic experiences. Dance therapy interventions lead to significant improvements in body perception, somatic awareness and self-worth (Allet et al., 2017; Malkina-Pykh, 2012; Muller-Pinget et al., 2012). At the beginning of the interventions, participants have limited access to proprioceptive and kinesthetic information and often focus primarily on visual or cognitive-emotional aspects. Through dance, they increasingly integrate bodily information, perceive their bodies as sources of meaning and action, and develop more positive body and self-image (Meekums et al., 2012; Muller-Pinget et al., 2018). This embodied learning simultaneously engages physical, emotional and psychological dimensions, making dance therapy particularly effective and often more acceptable than conventional exercise approaches for individuals with obesity (Muller-Pinget et al., 2012). The integration of movement, emotion, and imagination may contribute to emotional regulation processes.

While the dance interventions, perceptual patterns (visual and emotional), aesthetic preferences, and individual reference standards of participants shifted. Movements were increasingly evaluated in terms of expression and embodied experience rather than external norms, which contributed to more positive body evaluations (Malkina-Pykh, 2012). This may indicate a shift from an externally evaluated body perspective toward a more embodied experience of the body. The body was no longer experienced as a rigid mass but as a living, flowing subject characterized by refined sensorimotor functioning and expanded possibilities for action (Muller-Pinget et al., 2012, 2018). These embodied experiences generated immediate physical, emotional, and psychosocial effects, strengthened self-worth, and enabled a re-evaluation of movement and bodily experience, findings that are also supported by the review by Schwender et al. (2018). Malkina-Pykh (2012) further demonstrated that emotional expression functions as an adaptive coping strategy and supports increased self-esteem.

Another important mechanism is social resonance. Five studies emphasize the role of social interaction (Allet et al., 2017; Azevedo et al., 2013; Muller-Pinget et al., 2012, 2018, 2019), which promotes exchange, support, and a sense of belonging and positively influences bodily experience (Allet et al., 2017; Muller-Pinget et al., 2012). These findings indicate that culturally embedded group-based dance may influence body image and self-worth through mechanisms such as belonging, identity formation, and social connectedness. This dimension corresponds to self-determination theory, according to which relatedness enhances psychological well-being (Ryan and Deci, 2000) and supports connection to the self and the body. Social gaze is no longer perceived as threatening but rather as supportive, which can be partly explained by Polyvagal theory (Porges, 1991). Dance also opens new embodied and emotional experiential spaces that extend beyond the social possibilities of other movement-based interventions, such as yoga, which typically emphasize mindfulness or stress reduction and provide only limited opportunities for social interaction (Kishida et al., 2019).

The studies indicate that dance activates somatic, aesthetic, and reflective learning processes, which are considered central mechanisms of action (Allet et al., 2017; Malkina-Pykh, 2012; Muller-Pinget et al., 2012). Embodied self-perception forms the basis for self-reflection and enables transformations in body image (Schwender et al., 2025). By integrating cognitive and affective-emotional dimensions, dance promotes holistic reflective processes (Vocks et al., 2018) and supports the development of a conscious and appreciative relationship with the body (Grawe, 2000; Maslow, 2024). Dance is grounded in sensorimotor-reflective self-perception, performative practice, and experiential learning, where cognitive and emotional disturbances can act as triggers for transformative processes (Rudi, 2021; Schwender et al., 2025).

Overall, the findings highlight the transformative potential of dance: through embodied, aesthetic, and social processes, dance enhances body perception, emotional regulation, self-worth, and social connectedness. Thus, it addresses key aspects that are often neglected in conventional obesity interventions and represents a promising approach for the sustainable improvement of body image and self-worth.

### 4.2 Improvement of body image independent of weight loss

A key finding of this review is the improvement of body image through dance interventions independent of changes in body weight. This is particularly relevant because body image in individuals with obesity is often closely associated with (perceived) body weight and may promote negative self-evaluation (Muller-Pinget et al., 2019). Accordingly, many participants reported markedly impaired body image, high levels of dissatisfaction, and limited body perception at baseline (Akyilmaz et al., 2023; Carraça et al., 2012; Muller-Pinget et al., 2012, 2019).

Nearly all studies demonstrated improvements in body image following the intervention. Only one study reported no change, which was likely attributable to the relatively short intervention duration of 16 weeks (Muller-Pinget et al., 2018). At the same time, other findings indicate that even short interventions can produce measurable improvements in body image and bodily self-perception (Shetty et al., 2019). Overall, the results suggest that dance can foster processes of body-related self-perception.

These findings further suggest that dance strengthens body awareness and promotes processes of self-acceptance, psychological well-being, and self-worth (Carraça et al., 2012; Malkina-Pykh, 2012). These changes are often accompanied by a reduction in emotional eating behavior and indicate that, in addition to body-related effects, emotion-regulatory processes are also positively influenced. The findings may indicate a reciprocal relationship between body image processes and weight-related behaviors. Several studies show that these effects are not strictly dependent on weight reduction but are instead based on improved access to bodily sensations (Muller-Pinget et al., 2012).

The results contradict the assumption that changes in body image necessarily require prior weight reduction. Although previous studies show a close relationship between body image and body weight and a tendency for weight gain to negatively affect body image (Cash, 1994; Foster et al., 1997; Friedman et al., 2002; Pollatou et al., 2010; Schwartz and Brownell, 2004), several of the included studies did not find such an association (Malkina-Pykh, 2012; Muller-Pinget et al., 2012, 2019). Instead, individual progress, such as increased self-efficacy and positive bodily sensations, proves to be central (Carraça et al., 2012).

Thus, the subjective perception of one’s own performance level and bodily experience appears to contribute more strongly to improvements in body image than actual weight reduction. Consequently, the promotion of a positive body image and differentiated body awareness should constitute central components of obesity treatment, ideally as a complementary or even preparatory element to weight-reducing interventions (Muller-Pinget et al., 2012).

Although the majority of studies demonstrate improvements in body image independent of body weight, six studies nevertheless reported weight changes during the intervention period (Akyilmaz et al., 2023; Meekums et al., 2012; Muller-Pinget et al., 2012, 2018, 2019; Shetty et al., 2019). The role of weight reduction as a contributing mechanism remains unclear and requires further investigation. The simultaneous improvement of physical and psychological outcomes suggests that structured dance formats may address both appearance-related and functional aspects of body experience. This particularly concerns interventions within the “Aesthetics fitness and technique” component, in which structured Zumba and aerobic movement routines were applied. Notably, in the study by Meekums et al. (2012), despite comparable weight reduction, only the dance group showed additional improvements in emotional eating behavior, well-being, self-esteem, and body image. This suggests a specific effect of dance-based interventions on body experience and emotion regulation.

The findings also illustrate the ambivalent role of physical appearance: Negative body evaluations may be associated with and contribute to the maintenance of dysfunctional eating behavior (Friedman et al., 2002; Muller-Pinget et al., 2019; Schwartz and Brownell, 2004). At the same time, improvements in body image may help reduce such behavior. Dance thereby functions as a mediating mechanism between body perception, emotion regulation, and eating behavior (Carraça et al., 2012; Johnson and Wardle, 2005; Malkina-Pykh, 2012).

The results also raise questions regarding current developments such as the use of weight-reducing medications (e.g., anti-obesity injections) (Berg et al., 2025). Since body image can improve through dance independently of weight change, it remains questionable whether purely pharmacologically induced weight reduction without accompanying interventions targeting body image work will be sufficiently effective in the long term. Rather, the findings suggest that combined approaches may be necessary to prevent relapse (e.g., weight regain) or renewed deterioration of body image (Berg et al., 2025; Wilding et al., 2022). This suggests that dance interventions may influence body image through mechanisms beyond weight reduction.

### 4.3 Enhancement of self-worth through non-performance-oriented dance

The results show that dance-specific interventions strengthen self-esteem particularly in non-performance-oriented, aesthetically and culturally oriented settings (Schwender et al., 2018). In contrast to competitive sports contexts, they enable an experience-based, non-judgmental engagement with one’s own body, free from performance pressure and external evaluation.

This becomes especially evident in creative movement tasks. For example, the study by Muller-Pinget et al. (2018) shows that, among other elements, creative transformation exercises in which participants develop their own movements contribute significantly to the enhancement of self-esteem. These findings are consistent with (Fox, 1999), who attributes positive effects of physical activity on self-concept, body image, and well-being primarily to the subjective experience of movement.

A central finding is demonstrated in the study by Meekums et al. (2012): Only the dance intervention group, but not the control group, showed a significant increase in self-esteem. This underscores the specific effectiveness of dance-based interventions, which extends beyond mere physical activity and particularly incorporates expressive, reflective, and interpersonal dimensions. The absence of external evaluation allows participants to focus more strongly on their own bodily experience and emotional processes (Rudi, 2021; Steinberg and Rudi, 2024), thereby creating creative experiential spaces that promote self-perception. These findings are supported by Schwender et al. (2025), who describe creative dance settings as spaces of reflective practice in which bodily, emotional, and aesthetic experiences can be integrated. These settings foster both deeper bodily experience and processes of self-reflection and identity development (Akyilmaz et al., 2023; Azevedo et al., 2013; Carraça et al., 2012; Meekums et al., 2012; Muller-Pinget et al., 2012).

In addition to self-efficacy, studies also report an increase in self-acceptance during the course of dance-based interventions (Muller-Pinget et al., 2019, 2018). Participants reported improved acceptance of their body, personality, and individual expression (Muller-Pinget et al., 2018), which was associated with increased self-worth, greater self-confidence, and a positive basic attitude (Azevedo et al., 2013). Stable self-worth is considered a protective factor against negative psychological and physical consequences of obesity (Puhl et al., 2007).

At the same time, obesity research emphasizes the role of self-worth in the development, maintenance, and treatment of obesity. Hebebrand and Herpertz-Dahlmann (2009), Incledon et al. (2011), and Zametkin et al. (2004) highlight that low self-esteem can function as a risk factor for emotional eating behavior and may therefore influence the course of the disorder. The present findings suggest that dance-based interventions may specifically counteract these risk factors.

In summary, the results show that creativity- and expression-oriented dance interventions can make an essential contribution to the enhancement of self-esteem. They provide individuals with obesity with experiential access to their own body and thus represent a central component for sustainable and holistic treatment.

### 4.4 Heterogeneity of intervention content, consistent mechanism of action and sustainability

The studies included in the review demonstrate high methodological and conceptual heterogeneity with regard to research design, assessment methods, as well as the content, duration, and didactic-methodological focus of the interventions. Also the inconsistent terminology represents a conceptual challenge for synthesizing evidence and highlights the need for clearer theoretical differentiation in future research. As a result, it remains unclear which specific components are responsible for the observed positive effects on body image and self-esteem. At the same time, this diversity reflects the broad range of approaches through which dance interventions can be examined in individuals with obesity. The heterogeneity of intervention formats, measurement approaches, and samples prevented quantitative synthesis and limited the generalizability of conclusions.

Despite the limited number of studies, a consistent pattern of positive effects emerges. Randomized controlled dance interventions lead to significant improvements in evaluative and dysfunctional body image as well as self-esteem, in some cases with sustained effects beyond the intervention period (Akyilmaz et al., 2023; Allet et al., 2017; Carraça et al., 2012; Malkina-Pykh, 2012; Muller-Pinget et al., 2018, 2019; Shetty et al., 2019). Improvements in body perception and body dissatisfaction can be directly attributed to the interventions, whereas methodologically less robust studies provide only indications of similar developments.

The explanatory power of the findings remains limited due to methodological constraints, as only one study included a follow-up phase. Nevertheless, all studies show a consistent pattern of positive effects regardless of their primary focus, although the magnitude of effects varies depending on the research objectives and study design. The heterogeneity of intervention formats, measurement approaches, and samples further prevented quantitative synthesis and limits the generalizability of conclusions. This consistency despite limited evidence highlights the potential of dance to address central psychological and body-related factors that are often neglected in conventional obesity treatments.

## 5 Strengths, weaknesses, and recommendations

### 5.1 Strengths

The review demonstrates several methodological strengths. Adherence to the PRISMA guidelines ensures transparency and reproducibility throughout the review process. Clearly defined inclusion and exclusion criteria allowed for the inclusion broad range of studies, despite the overall limited research base on dance-based interventions in obesity. The literature searches were conducted across multiple databases, and three independent reviewers were involved in the screening and selection process. The assessment of study quality was based on the “Checklist for Qualitative Research” (Lockwood et al., 2015) and the “Checklist for Randomized Trials” (Barker et al., 2023). As a limitation, it should be noted that the search was restricted to English- and German-language journal articles, which may have resulted in the omission of relevant studies.

Nevertheless, the Wheel of Dance provides a theoretically grounded and practice-oriented framework for analyzing the included studies. Despite the heterogeneity of interventions, this model facilitates the identification of commonalities across studies, the exploration of potential mechanisms of action, and the derivation of preliminary implications for dance education research and practice. Particularly in the context of small sample sizes and heterogeneous study designs, the model proves to be a useful analytical tool, as conventional comparative methods are only of limited applicability.

### 5.2 Weaknesses

Despite the aforementioned strengths, this review is subject to several limitations. As noted by Andrews (2005), potential sources of bias in the search process, study selection, and synthesis can be reduced but not entirely eliminated. Consequently, completeness and absolute objectivity cannot be guaranteed. However, adherence to the PRISMA guidelines contributes to ensuring transparency, validity, and reproducibility to the greatest extent possible.

Another limitation is the small number of included studies, which restricts the overall strength of the evidence base. In addition, the considerable heterogeneity of study designs further limits comparability across studies. Differences in sample size, intervention duration, methodological approaches, and outcome measures hinder direct comparison and allow only cautious conclusions regarding potential mechanisms and effects of dance-based interventions. Taken together, these limitations highlight the need for further research and underscore the importance of expanding the evidence base through methodologically comparable and high-quality studies.

### 5.3 Recommendations for future research and practice

The results demonstrate that dance has considerable potential to improve body image and self-esteem in individuals with obesity. For future research, a more differentiated analysis of individual intervention components is necessary in order to systematically identify specific mechanisms of action as well as potential advantages and disadvantages and to enable a differentiated classification of the effects of dance-specific interventions. Furthermore, future research should place greater emphasis on conceptual clarity regarding key psychological constructs such as *self-worth* and *self-esteem*, as their inconsistent use currently further complicates the comparability of study findings.

First, future interventions should be developed in a stronger theory-driven manner, understanding dance as an aesthetic-cultural practice (Steinberg and Rudi, 2024) and grounding it in sensorimotor differentiation, emotional expression, and social resonance. Relevant theoretical models, such as embodied cognition and education, somatic education, and body-related experiential learning, should be integrated from the outset (Francesconi and Tarozzi, 2019; Koch, 2011; Schwender et al., 2025; Singh and Narayanan, 2021), while creative, non-performance-oriented elements that show particularly strong effects should be clearly described and justified.

Second, the pronounced heterogeneity of previous studies requires more precise documentation of intervention content. Future work should systematically describe core components such as improvisation, body awareness exercises, choreographic tasks, and group interactions, including didactic decisions, handling of performance demands, and the weighting of somatic, creative, emotional, and social elements. This will enable more targeted identification of mechanisms of action.

Third, intervention effects should be assessed using both subjective and objective measures. Changes in body image and self-esteem are closely linked to embodied perception and behavioral patterns; therefore, the use of validated measurement instruments as well as qualitative methods such as interviews, diary analyses, or movement observations is recommended. This can additionally capture which intervention aspects are experienced as meaningful from the participants’ perspective, supporting the further development of intervention design. Mixed-methods designs enable a comprehensive assessment of relevant processes and participant feedback. Purely quantitative designs cannot capture such insights.

A central aspect concerns the orientation and design of interventions as well as their systematic documentation and analysis. The review results show that social dynamics, such as group belonging, mutual recognition, and shared movement experiences, significantly influence body-related attitudes in a positive way. Future studies should therefore assess these social dimensions and document contextual conditions such as group size, interaction forms, and familiarity among participants.

Furthermore, non-performance-oriented, creativity-promoting dance formats appear particularly effective, as they reduce performance pressure, emphasize individual expression opportunities, and support reflective learning processes. A clear description of the didactic design in relation to study design and outcome measures is essential for the transparency and further development of future interventions.

Finally, dance should be more strongly integrated into therapeutic and health-psychological contexts, as it addresses and transforms physical, emotional, and psychosocial dimensions simultaneously.

## 6 Summary

The aim of the systematic literature review was to identify studies examining the effects of dance interventions on body image and self-worth. Overall, the analysis of the ten included studies suggests that dance interventions may have positive effects on body image and self-worth, emotion regulation, and quality of life, particularly in contexts where conventional physical activities reach their limits, such as in deeply rooted patterns like alexithymia. However, given the limited number of studies and the methodological heterogeneity of the included investigations, these findings should be interpreted with caution.

Nevertheless, the findings highlight the potential relevance of dance-based interventions for physical education and developmental contexts, as well as for health-related and (sport-)pedagogical settings. Formats derived from dance and movement therapy are characterized by aesthetic experience, body awareness, and subjective expression, making their underlying principles at least partially transferable to pedagogical dance instruction.

A key mechanism appears to the shift away from normatively structured movement requirements toward individually experienced, sensory-rich movement process, as well as an object of evaluation and performance toward the body as a subjectively experienced and acting lived body. In dance-pedagogical contexts, this may create experiential spaces that foster bodily awareness and social resonance.

Consequently, dance-based interventions should be designed with clear didactic intention: depending on the intended outcomes, such as psychosocial strengthening, aesthetic experience, or physical-motor development, appropriate teaching formats, task designs, and reflective components should be selected. Elements such as non-performance-oriented approaches, creative openness, and social inclusion, may also be transferable to other educational contexts, such as physical education in schools.

For future research, there is a need to systematically examine the sustainability of dance-specific effects, to compare different dance styles and instructional approaches, and to further integrate psychological and pedagogical perspectives in order to better evaluate their potential in additional contexts such as school sports or community-based programs. Overall, the existing evidence suggests that dance as an aesthetic-cultural practice may hold potential for fostering embodied learning and developmental processes; however, robust conclusions require further methodologically rigorous and comparable studies.

## Data Availability

The original contributions presented in this study are included in the article/supplementary material, further inquiries can be directed to the corresponding author.

## Footnotes

1
https://www.crd.york.ac.uk/PROSPERO/view/CRD42024604272

2 https://jbi.global/sites/default/files/2019-05/JBI_Critical_Appraisal-Checklist_for_Qualitative_Research2017_0.pdf (Qualitativ Research).

3 https://journals.lww.com/jbisrir/fulltext/2023/03000/the_revised_jbi_critical_appraisal_tool_for_the.5.aspx (Randomized Controlled Trials).
